# I spy with my MRI: Cause of complete heart block in a young patient revealed

**DOI:** 10.1007/s12471-017-0997-9

**Published:** 2017-04-26

**Authors:** R. P. Amier, R. B. van Loon, L. J. Meijboom, R. Nijveldt

**Affiliations:** 1 Department of Cardiology, VU University Medical Center (VUmc), Amsterdam, The Netherlands; 2 Department of Radiology, VU University Medical Center (VUmc), Amsterdam, The Netherlands

**Fig. 1:**
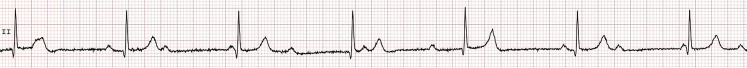
ECG showing third-degree atrioventricular block with a junctional escape rhythm of 45 bpm

**Fig. 2:**
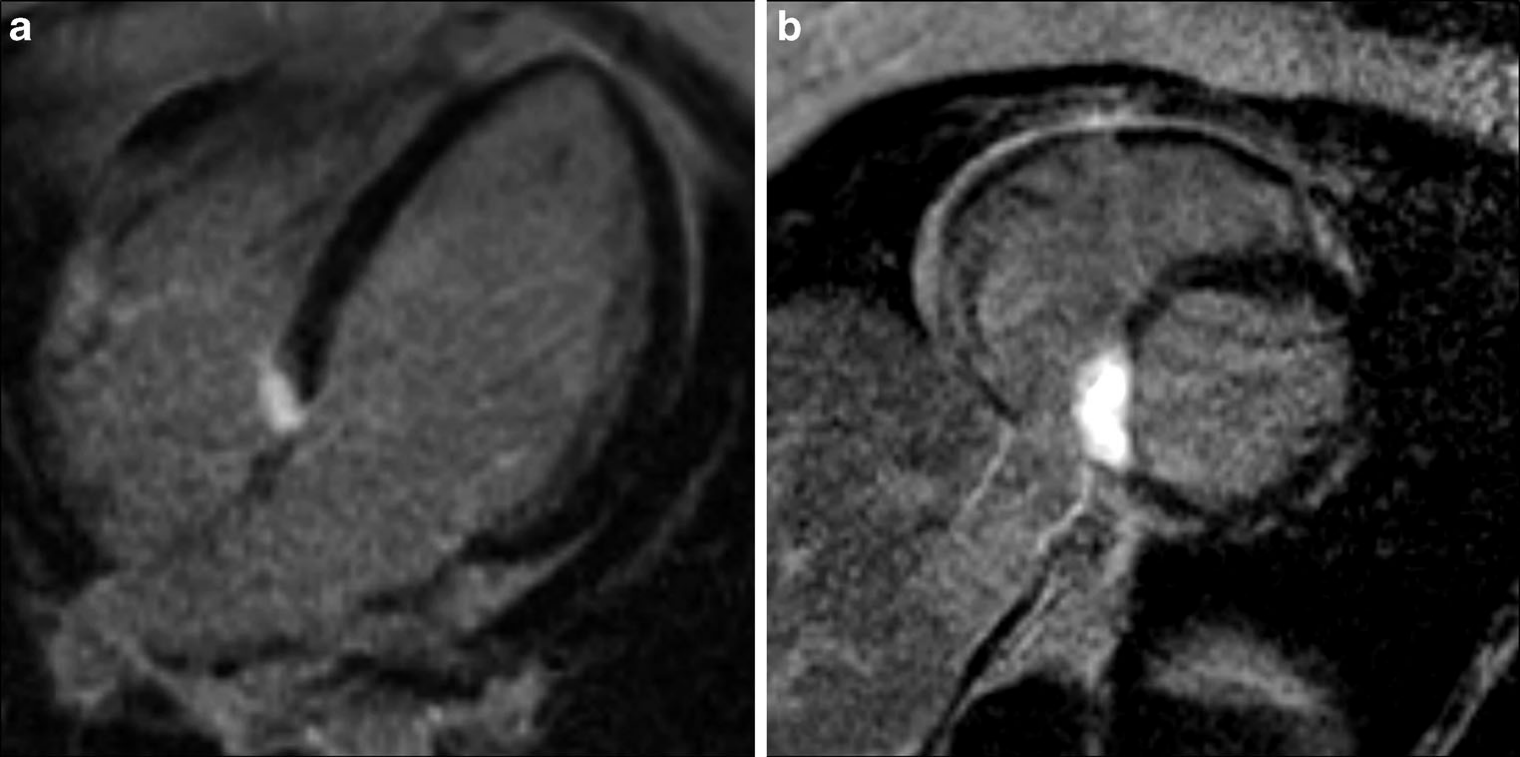
LGE-CMR images showing basal inferoseptal fibroma in (**a**) 4-chamber view and (**b**) short-axis view

A 21-year-old female patient presented with unexplained weight gain and dyspnoea on exertion. The ECG showed third-degree atrioventricular (AV) block with a junctional escape rhythm of 45 bpm (Fig. 1). Echocardiography showed no cardiac abnormalities. However, subsequent CMR using late gadolinium enhancement (LGE) revealed a single region (32 × 15 mm) of intramyocardial, high-intense, homogeneous contrast enhancement with a well-defined border in the basal inferoseptal myocardium (Fig. 2). Additional sequences ruled out the presence of fat. The septal localisation, homogeneous high-intense contrast enhancement with smooth borders, in the absence of fat, pericardial effusion or systemic disease such as sarcoidosis, all attest to an intramyocardial fibroma as the most likely diagnosis. The fibroma is thought to impede the conductive system, in this case leading to third-degree AV block. Cardiac fibromas are benign congenital tumours with predominant localisation in the ventricular septum and left ventricular (LV) free wall, where tumour mass effect can cause arrhythmias, haemodynamic obstruction, heart failure or even sudden cardiac death. Fibromas do not regress spontaneously, therefore monitoring of growth is indicated. Treatment can include pacemaker implantation for arrhythmias and surgical excision in case of LV outflow obstruction. This case exemplifies the crucial value of tissue characterisation by LGE-CMR in identifying a cardiac fibroma causing heart block in a young patient.

